# From astrocytoma to glioblastoma: a clonal evolution study

**DOI:** 10.1002/2211-5463.12815

**Published:** 2020-03-22

**Authors:** Fuhua Yang, Yunding Zou, Qiang Gong, Jieping Chen, Wei‐Dong Li, Qilin Huang

**Affiliations:** ^1^ Department of Genetics School of Basic Medical Sciences Tianjin Medical University China; ^2^ Tianjin Key Laboratory of Retinal Functions and Diseases Eye Institute and School of Optometry Tianjin Medical University Eye Hospital China; ^3^ Department of Hematology Southwest Hospital The Army Medical University Chongqing China; ^4^ Department of Neurosurgery Xinqiao Hospital The Army Medical University Chongqing China

**Keywords:** astrocytoma, clonal evolution, glioblastoma, relapse, TP53

## Abstract

Astrocytomas often recur after surgical resection, but the underlying mechanism remains enigmatic. Elucidation of clonal evolution in primary and relapse tumors may provide important information on tumor progression. Here, we examined genetic factors underlying recurrence in a patient with astrocytoma initially diagnosed with World Health Organization (WHO) grade II astrocytoma, who then relapsed with glioblastoma (WHO grade IV) complicated with local anaplastic astrocytoma (WHO grade III). We performed genomic DNA sequencing and data analysis of paired tumor tissue specimens and a peripheral blood sample (control), and used expands software for subclone analysis. A germline *NOTCH1* missense mutation was identified in the peripheral blood sample, the primary tumor and the relapse tumor; in addition, we identified a *tumor protein p53* (*TP53*) heterozygous nonsense mutation in the primary tumor and a *TP53* homozygous nonsense mutation and an *IDH1* heterozygous missense mutation in the relapse tumor. Clonal evolution trees indicated higher heterogeneity in the relapse tumor. Although germline mutations might contribute to the driving force of the primary tumor, aggressive chemotherapy and radiation may apply selective pressure for tumor clonal evolution; furthermore, a total loss of function of gatekeeping genes (*TP53*) may result in impaired DNA repair and catastrophic chromosomal aberrations.

## Graphical abstract

Astrocytoma often recurs after surgery. In this study, we examined the clonal structures of a primary astrocytoma and its relapse tumor, a malignant glioblastoma. We observed accelerated carcinogenesis in the relapse tumor after intensive radiation and chemotherapy, suggesting that such treatment may contribute to astrocytoma malignant progression.

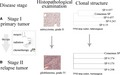

**Abbreviations**
BWA: burrows‐wheeler aligner
CNV: copy number variation
IDH: isocitrate dehydrogenase
INDELs: insertions and deletions
SNP: single nucleotide polymorphism
SP: subpopulation
SV: structure variation
TP53: tumor protein p53
WHO: World Health Organization

Gliomas are the most common central nervous system tumors derived from glial cells. Traditionally, they are classified according to the World Health Organization (WHO) and include astrocytomas, oligodendrogliomas and ependymomas [1]. Like most cancers, gliomas develop because of genetic aberrations that accumulate with tumor progression [2, 3, 4]. Astrocytomas are tumors that arise from astrocyte cells that make up the “glue‐like” or supportive tissues of the brain. Low‐grade astrocytomas are usually localized and grow slowly; high‐grade astrocytomas grow at a rapid pace and require a different course of treatment.

Currently, the treatment options for astrocytoma include surgery, radiation and chemotherapy. Compared with high‐grade astrocytomas, low‐grade astrocytomas are slow growing, but over time they may progress to more malignant tumors after resection, leading to reduced overall survival. The factors associated with recurrence and malignant degeneration vary and include patient age, tumor size, type, location and the extent of resection [5, 6, 7, 8, 9]. The genetic factors were also proved to play an important role in the pathogenicity and development of gliomas disease. Most cases of astrocytoma‐associated mortality are due to tumor recurrence and malignant transformation, which may be associated with clonal evolution at the cytogenetic level [10, 11, 12, 13]. Over about the past 10 years, the number of studies focused on the molecular biology of astrocytomas has increased significantly. Numbers of genetic markers associated with astrocytomas were identified [14, 15, 16, 17]. Studies have shown that low‐grade astrocytomas usually carry mutations of *isocitrate dehydrogenase 1/2* (*IDH1/2*) and *tumor protein p53* (*TP53*) gene [18, 19, 20]. In contrast, studies show that glioblastoma usually arises without *IDH* mutation [19]. Research data also identified different genetic changes associated with the degree of astrocytomas differentiation [21, 22]. To determine the genetic spectrum associated with relapse and malignant transformation of astrocytoma, we performed whole‐genome sequencing of primary tumor, relapse tumor, and a peripheral blood sample from a patient first diagnosed with grade II astrocytoma who then relapsed with glioblastoma (grade IV) complicated by local anaplastic astrocytoma (grade III).

## Materials and methods

### Patient and samples

A 25‐year‐old female patient was diagnosed with grade II astrocytoma with accompanying secondary epilepsy, and surgery was conducted to remove the tumor tissues, followed with radiation (60 Gy total) and chemotherapy (nimustine, 80 mg·m^−2^, 1 week × 8). Then, after almost 1 year, the tumor recurred, and histopathological examination of the resected specimen revealed that the tumor was glioblastoma (grade IV) complicated by local anaplastic astrocytoma (grade III). The patient was treated by temozolomide (120 mg·m^−2^, days 1–5 every 28 days for 5 cycles).

Samples of primary (coded as TY‐1) and relapse tumor (TY‐2) and a peripheral blood sample (TY‐NC) were collected, and genomic DNA was extracted from all samples using standard protocols. Then the DNA samples were cryostored for whole‐genome sequencing. The patient gave written informed consent, and the Committee on Studies Involving Human Beings at Army Medical University approved the protocol. The research complied with the Helsinki Declaration and International Ethical Guidelines for Biomedical Research Involving Human Tissue, jointly developed by the WHO and the International Council of Medical Science Organizations.

### Whole‐genome sequencing

Adequate amounts of high‐quality DNA of tumor and normal samples were used to construct libraries (TruSeq Library Construction Kit; Illumina, San Diego, CA, USA) according to the manufacturer’s supplied protocols. High qualified DNA samples were randomly broken into 350‐bp fragments using ultrasonicator. Then the fragmented genomic DNA was end repaired and phosphorylated, followed by the addition of A‐tailing, ligated index adaptation, denaturing and amplification for the final product. After genomic DNA library detection, the qualified libraries were sequenced using HiSeq XTen (Illumina). The depth of sequencing was 100× for tumor samples and 30× for the peripheral blood sample.

### Bioinformatics analyses of the sequence data

The raw data (sequenced reads) acquired by sequencing were preprocessed by eliminating the low‐quality reads. The sequence coverage for the primary and relapse tumor samples was 98.85% and 98.68%, respectively. The cleaned reads of tumor and normal tissues were then aligned to reference genome (UCSU hg19) [23] using burrows‐wheeler aligner (BWA) software [24] to obtain the initial results, saved in BWA format. The BWA format files were processed by duplication removal, local realignment and base quality recalibration using picard (http://sourceforge.net/projects/picard/), gatk [25] and samtools [26], respectively, to get the final comparison results. Single nucleotide polymorphisms (SNPs) and insertions and deletions (INDELs) were detected using gatk, whereas copy number variations (CNVs) were identified by control‐FREEC [27]. Then variations were annotated using ANNOVAR [28], including genes related to variation, genomic character annotation and function of related genes. Then the somatic SNPs and INDELs were identified using mutect [29] and strelka [30] software, whereas somatic structure variations (SVs), including interchromosomal and intrachromosomal translocations, deletions and insertions, were identified using crest [31], and somatic CNVs were identified by control‐FREEC (Fig. S1). The feature of the identified variants was visualized using the Integrative Genomics Viewer tool [32].

### Clonal evolution analysis

Clonal evolution analysis of the primary and the relapse tumor was conducted using expands [33]. Based on the somatic SNPs and CNVs of the tumor, expands predicts the number of subpopulations (SPs) that coexist in a tumor, the size of the SPs in the tumor bulk and the mutations that mark each SP.

## Results

### SNPs, INDELs, CNVs and chromosome translocations detected in primary and relapse tumor by whole‐genome sequencing

**Fig. 1:**
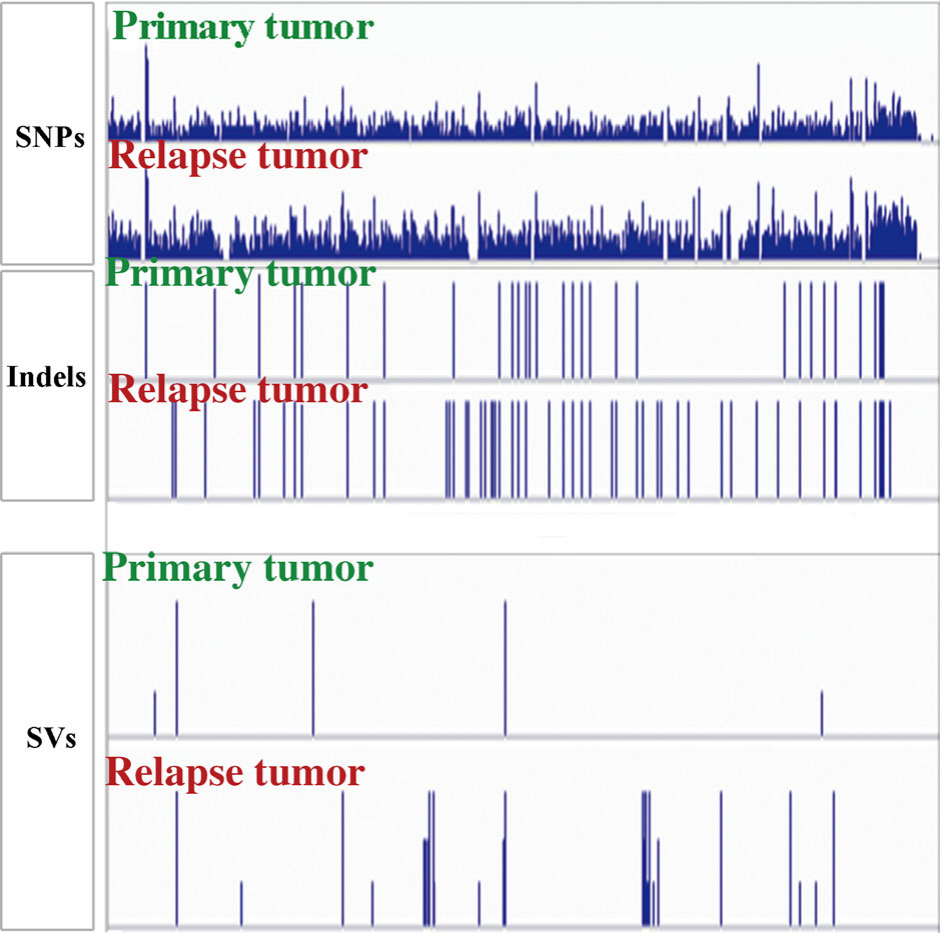
Genetics variants detected in primary and relapse tumor tissues. The identified variants were displayed using the Integrative Genomics Viewer.

**Fig. 2:**
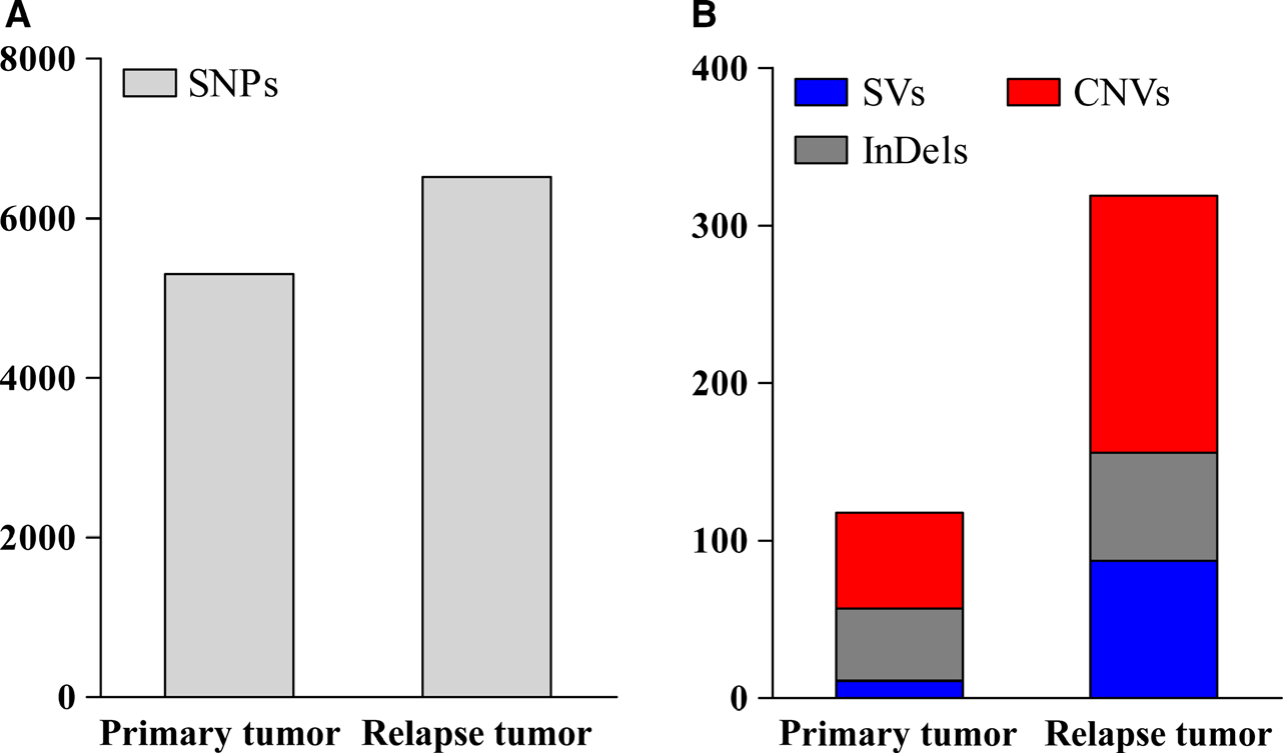
Numbers of SNPs (A), SVs (B), CNVs (B) and insertion and deletions (InDels; B) in the primary and relapse tumor. After filtering the low‐quality reads data, the clean data were used to identify the genetic variants; more variants were detected in the relapse tumor.

Mutations of primary tumor and relapse tumor were detected through aligning the sequence data to the reference genome (UCSC hg19) (Fig. 1). Compared with the primary tumor, more genetic variants (including the SNPs, INDELs, CNVs and chromosome translocations) were detected in the relapse tumor (Fig. 2). Amino acid–altering SNP annotations were conducted, including the SNPs located in cancer‐related genes. The cancer‐related genes in the peripheral blood sample and primary and relapse tumors are listed in Table 1.

**Table 1:** Amino acid–altering sequence variants located on cancer‐related genes in the peripheral blood sample, primary tumor and relapse tumor. 0/1, heterozygote; 1/1, homozygote; GT, genotype.

Sample	Gene	snpID	Variant	GT
Peripheral blood	*NOTCH1*	–	NC_000009.11:g.139408966T>C NM_017617:exon13:c.A2203G:p.N735D	0/1
Primary tumor	*ATIC*	rs2372536	NC_000002.11:g.216190020C>G NM_004044:exon5:c.C347G:p.T116S	0/1
	*CIITA*	rs7197779	NC_000016.9:g.11002927A>G NM_001286403:exon10:c.A947G:p.Q316R	1/1
	*NOTCH1*	–	NC_000009.11:g.139408966T>C NM_017617:exon13:c.A2203G:p.N735D	0/1
	*KCNJ5*	rs7102584	NC_000011.9:g.128782012C>G NM_000890:exon2:c.C844G:p.Q282E	1/1
	*PDE4DIP*	rs140993521	NC_000001.10:g.144863438G>T NM_001198834:exon37:c.C5965A:p.Q1989K	0/1
		rs1698605	NC_000001.10:g.144871738C>A NM_001198834:exon32:c.G5224T:p.A1742S	0/1
		rs145568299	NC_000001.10:g.144922593C>T NM_001002811:exon3:c.G1303A:p.A435T	0/1
	*PER1*	rs2585405	NC_000017.10:g.8046772C>G NM_002616:exon19:c.G2884C:p.A962P	0/1
	*ROS1*	rs619203	NC_000006.11:g.117622184G>C NM_002944:exon42:c.C6686G:p.S2229C	0/1
		rs529156	NC_000006.11:g.117622188T>G NM_002944:exon42:c.A6682C:p.K2228Q	0/1
		rs529038	NC_000006.11:g.117622233C>T NM_002944:exon42:c.G6637A:p.D2213N	0/1
	*TP53*	–	NC_000017.10:g.7578492C>T NM_001126115:exon1:c.G42A:p.W14X	0/1
Relapse tumor	*IDH1*	rs121913500	NC_000002.11:g.209113112C>T NM_001282386:exon4:c.G395A:p.R132H	0/1
	*NOTCH2*	rs11810554	NC_000001.10:g.120611964G>C NM_001200001:exon1:c.C57G:p.C19W	0/1
	*PDE4DIP*	rs2455986	NC_000001.10:g.144852390C>T NM_001198834:exon44:c.G7053A:p.W2351X	0/1
	*NOTCH1*	–	NC_000009.11:g.139408966T>C NM_017617:exon13:c.A2203G:p.N735D	0/1
	*PER1*	rs2585405	NC_000017.10:g.8046772C>G NM_002616:exon19:c.G2884C:p.A962P	1/1
	*TP53*	–	NC_000017.10:g.7578492C>T NM_001126115:exon1:c.G42A:p.W14X	1/1

### SPs detected in the primary and the relapse tumor

**Fig. 3:**
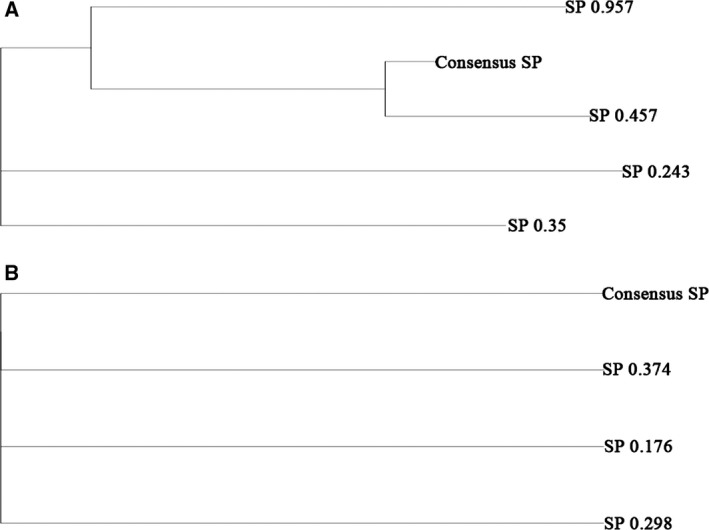
Clonal structures of the primary tumor (TY‐1, A) and the relapse tumor (TY‐2, B) constructed by expands. Somatic SNPs and CNVs were used to construct the clonal structures. A “tree‐like” structure was identified in the primary tumor (A) and a “parallel” structure in the relapse tumor (B).

Somatic mutations are gene mutations that occur in somatic cells after conception, which can lead to a variety of medical issues and are associated with cancers. Based on the somatic SNPs and CNVs of the tumor, four and three SPs were detected in the primary and the relapse tumor, respectively (Fig. 3). Dominant SPs detected in the relapse tumor shared no significant fraction of SNPs with SPs from the primary tumor.

### Somatic structural variants shared in the primary and the relapse tumor

Structural variants are large gene fragment variations in the genome that may influence the expression of genes whose biological functions are vital. First, the chromosome aberrations generated by the SVs were identified in the primary and relapse tumor, and then the genes involved in the chromosome variants were annotated. Chromosome aberrations involving the cancer‐related genes and/or DNA repair genes were shown in both primary and relapse tumor tissues (Table 2).

**Table 2:** Chromosome aberrations involving the cancer‐related genes and/or DNA repair genes in the primary tumor (astrocytoma, TY‐1) and the relapse tumor (glioblastoma, TY‐2).

Sample	Gene_A	Junction_A	Gene_B	Junction_B	Fusion pair
Primary tumor	*MRPL57*	13:21750661	*DDX10*	11:108585751	*MRPL57‐DDX10*
	*MYO5B*	18:47400964	*RAD54B*	8:95409470	*MYO5B‐RAD54B*
Relapse tumor	*BRIP1*	17:59932940	*ZNF529*	19:37039340	*BRIP1‐ZNF529*
	*NSD1*	5:176672875	*ROBO1*	3:79308398	*NSD1‐ROBO1*
	*BCOR*	X:39939333	*NPAS3*	14:33520196	*BCOR‐NPAS3*
	*PCM1*	8:17814538	*MLLT11*	1:151035150	*PCM1‐MLLT11*
	*SET*	9:131457148	*DPP10*	2:116376668	*SET‐DPP10*
	*ABL1*	9:133751372	*CFAP36*	2:55760693	*ABL1‐CFAP36*
	*FOXP1*	3:71030047	*C8orf37‐AS1*	8:96526713	*FOXP1‐C8orf37‐AS1*
	*ACSS2*	20:33462999	*RNF43*	17:56455089	*ACSS2‐RNF43*
	*LAMA1*	18:7073839	*PTPN11*	12:112866611	*LAMA1‐PTPN11*
	*FAM219A*	9:34453196	*NTRK3*	15:88462594	*FAM219A‐NTRK3*
	*SKA3*	13:21750661	*DDX10*	11:108585748	*SKA3‐DDX10*
	*PELI2*	14:56606684	*BRAF*	7:140486815	*PELI2‐BRAF*
	*LOC101927967*	2:78504999	*BCL11B*	14:99701952	*LOC101927967‐BCL11B*
	*EPB41L4B*	9:111981592	*ALK*	2:29974050	*EPB41L4B‐ALK*
	*RABGAP1L*	1:174333695	*MLH3*	14:75499567	*RABGAP1L‐MLH3*

## Discussion

Sequencing the genomic DNA of diverse cancers has revealed that intratumors have spatially and temporally heterogeneous clonal architecture [34, 35, 36, 37], which has clinical implications and contributes to therapy resistance [38, 39, 40]. Astrocytoma is the most common of the gliomas, and low‐grade astrocytomas could progress to malignancy. To reveal the pathogenesis and progression of astrocytomas, more studies have focused on identifying the genetic variants in different stages of the tumor. It is important to clarify the reasons why low‐grade tumors recur and progress to malignancy, because this dramatically shortens patient survival. Here we sequenced the genomic DNA of primary tumor and relapse tumor tissues from a patient with astrocytoma to explore the mutational profile associated with relapse, which allowed us to define clonal evolution patterns at relapse.

In this study, the “baseline” background of the patient was a heterozygous *NOTCH‐1* (NC_000009.11:g.139408966T>C) missense mutation (Table 1). Notch‐1 plays an essential role of cell fate determination. It is difficult to determine whether the astrocytoma cells originated from the *Notch‐1* mutation, although *Notch‐1* mutations are among driver mutations in all astrocytomas and glioblastomas, as well as normal tissues.

*IDH1* and *IDH2* genes mutations were hallmarks of gliomas [41]. *IDH1* gene mutation could be identified in most of the patients with low‐grade (grade II–III) astrocytomas and glioblastomas, whereas the gene mutation was rarely detected in patients with primary glioblastomas [18, 19, 42]. Although the *IDH1* mutation has been considered a hallmark of astrocytoma and a good prognosis, an *IDH1* mutation was not identified until the late stage (relapse tumor, WHO IV). In this case, the *IDH1* mutation is likely a secondary change after the accelerated growth of tumor cells and hypoxia.

*TP53* mutations were found in most of the patients with astrocytomas with *IDH1* mutations [43]. *TP53* mutations could be found in most of the low‐grade astrocytomas and secondary glioblastomas, in which the mutation rate in primary glioblastomas was relatively low [16, 20]. In our study, a heterozygous nonsense mutation of *TP53* (NC_000017.10:g.7578492C>T) was found in the astrocytoma (WHO II, TY‐1) sample. One of the key mutations found in the WHO stage IV glioblastoma is the homozygous *TP53* nonsense mutation. TP53 is a gatekeeper of cell survival after DNA damage; astrocytoma cells with the *TP53* mutation have an advantage under selection from radiation and chemotherapy. Indeed, the clonal structure changed dramatically from a “tree‐like” structure in astrocytoma to a “parallel” structure in glioblastoma, suggesting a multiple clonal origin after the *TP53* checkpoint failed.

The relapse of astrocytoma is quite common. Because surgical treatments are unlikely to remove all tumor cells, radiation and chemotherapy are suggested. However, very aggressive radiation (60 Gy) provides selection pressure for *TP53* mutation‐free cells: cells with *TP53* mutations and impaired TP53 function are more likely to survive after radiation therapy.

Indeed, the number of mutations and structure variants increased significantly in the relapse tumor. The tree‐like structure of subclones in the primary tumor could no longer be found in the relapse tumor, suggesting a much higher heterogeneity. The complete loss of *TP53* could result in an impaired repairing of DNA damage; therefore, DNA mutations and chromosomal rearrangement became uncontrollable.

As well known, both radiation and chemotherapy could induce DNA damage and mutations. While killing most of the tumor cells, chemotherapies and radiation may provide selection pressures on some of the tumors. It might be a little premature to blame radiation and chemotherapy for the notorious relapsing of glioblastoma, but aggressive radiation therapy of astrocytoma may need reappraisal.

## Conclusions

The progressive development from astrocytoma to malignant glioblastoma remains enigmatic; however, our clonal evolution studies suggest accelerated carcinogenesis in the relapse tumor after intensive radiation and chemotherapy. It may suggest that aggressive treatment may not be a good choice for astrocytoma.

## Conflict of interest

The authors declare no conflict of interest.

## Author contributions

JC, W‐DL and QH designed the study. FY, QH and W‐DL wrote the manuscript. YZ, QG and QH collected the subject and clinical data. FY and W‐DL analyzed the data.

## Supporting information


**Fig. S1.** The workflow for the experimental processing and data analysis.
