# Supplementary figures and images for: From astrocytoma to glioblastoma: a clonal evolution study
Source: FEBS Open Bio. 2020 Mar 22;10(5):744–51. doi: 10.1002/2211-5463.12815 (PMC7193157; doi:10.1002/2211-5463.12815)

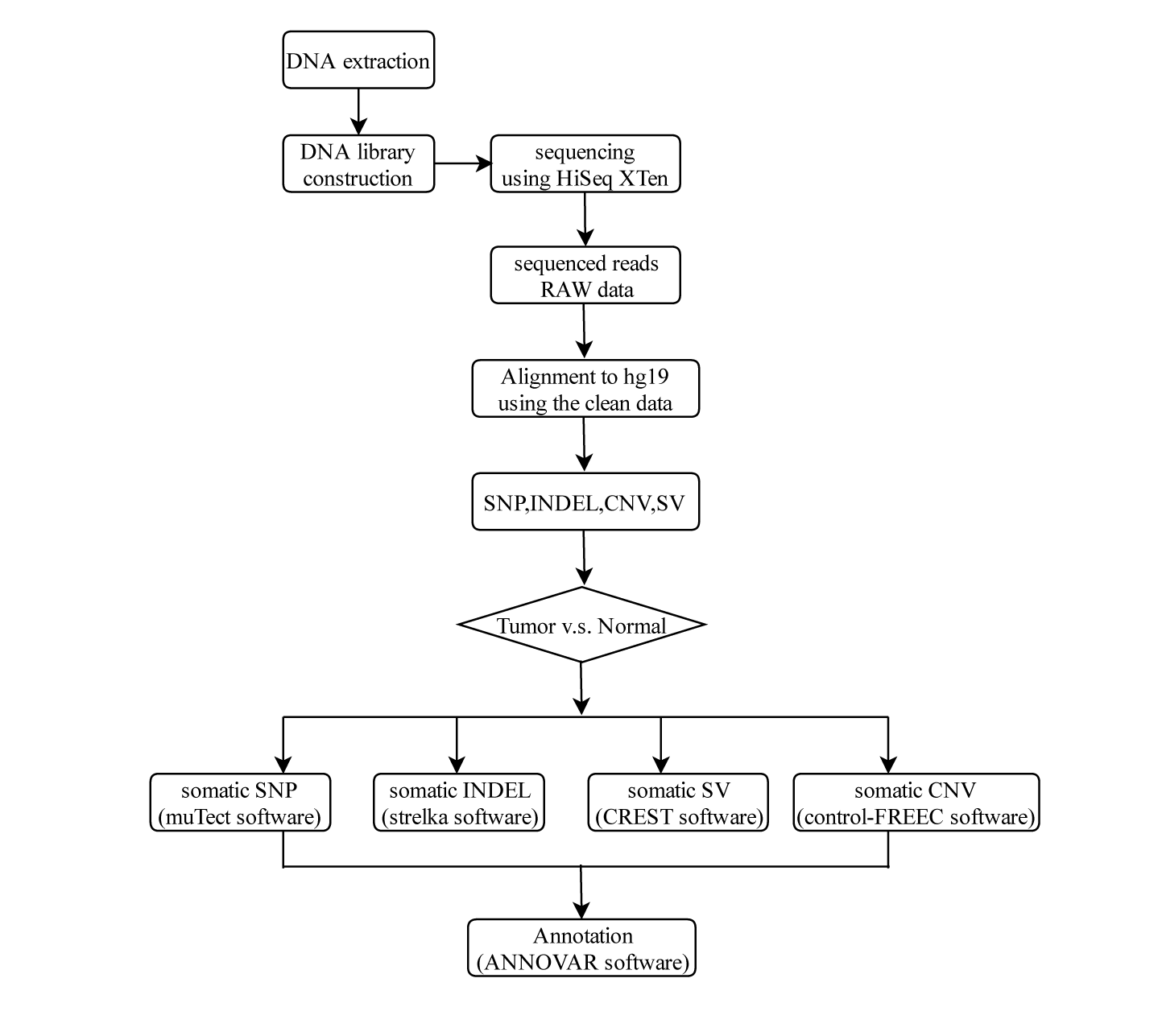

Supplement: Supplementary file 1 — Fig. S1. The workflow for the experimental processing and data analysis. [file FEB4-10-744-s001.tif]
